# Flexion Relaxation Ratio Not Responsive to Acutely Induced Low Back Pain from a Delayed Onset Muscle Soreness Protocol

**DOI:** 10.1155/2013/617698

**Published:** 2013-02-25

**Authors:** Maggie E. Horn, Mark D. Bishop

**Affiliations:** Department of Physical Therapy, University of Florida, P.O. Box 100 154, Gainesville, FL 32610, USA

## Abstract

*Background*. The flexion relaxation ratio (FRR) has been suggested as a measure of muscular performance in patients with low back pain (LBP). The purpose of this study was to investigate whether the FRR was responsive to acute LBP produced from a delayed onset muscle soreness (DOMS) protocol. *Methods*. Fifty-one pain-free volunteers performed DOMS to induce LBP. Current pain intensity, trunk flexion range of motion (ROM), and passive straight leg raise (SLR) were measured at baseline, 24 and 48 hours after DOMS. Participants were categorized into pain groups based on reported current pain intensity. Changes in FRR, trunk flexion ROM, and SLR ROM were examined using two-way repeated measures analysis of variance. *Results*. Pain group was not found to have a significant effect on FRR (*F*_1,29_ = 0.054, *P* = 0.818), nor were there any two-way interactions for changes in FRR. The pain group had decreased trunk flexion ROM compared to the minimal pain group (*F*_1,38_ = 7.21, *P* = 0.011), but no decreases in SLR ROM (*F*_1,38_ = 3.51, *P* = 0.057) over time. *Interpretation*. There were no differences in FRR based on reported pain intensity of LBP from a DOMS protocol. The responsiveness of FRR might be limited in patients with acute onset LBP of muscular origin.

## 1. Introduction

During normal trunk flexion in standing the trunk extensor muscles act eccentrically and are considered myoelectrically “active” until a distinct point in the trunk flexion range of motion (ROM) where the lumbar paraspinals relax. Passive structures and pelvic musculature provide the functional limit to terminal flexion ROM. During this time of “relaxation,” the paraspinal muscles of the trunk are considered myoelectrically “quiet” or electrically reduced. This process may otherwise be described as the flexion relaxation phenomenon or flexion relaxation. The flexion relaxation ratio (FRR) is a measure of muscle performance during the flexion relaxation phenomenon that can be quantified by the ratio of lumbar paraspinal muscle activity in full trunk flexion (relaxation) to the lumbar paraspinal muscle activity either during the flexion phase or extension from full flexion to upright stance using electromyography (EMG) [1]. 

The flexion relaxation phenomenon is often absent in persons experiencing chronic low back pain (LBP). In this population, lumbar paraspinal muscle activity actually increases during full flexion, particularly in the relaxation period where there is usually myoelectric silence in those experiencing chronic low back pain. Subsequently, changes in the FRR as measured by EMG occur based on this change in muscular activity. Investigators were previously able to correctly categorize 89% of subjects using the FRR into those experiencing chronic LBP and healthy controls [2]. Additionally, the FRR has been found to have high test-retest reliability in both pain-free subjects and those with low back pain [2, 3] and the FRR has been shown to be responsive to clinical status [4]. Neblett et al. (2003) followed patients with chronic LBP over a seven-week rehabilitation program. These investigators reported that 10 of 47 (21.3%) patients with LBP showed normal flexion relaxation prior to treatment. After treatment, 94% of patients who completed the program had normal flexion relaxation. Based on these findings, Colloca and Hinrichs (2005) have stated that the FRR can be used as clinical tool to aid in the diagnosis and treatment of patients with chronic LBP [5]. 

These authors also suggested that that the muscular changes resulting in the absence of FRR in persons with back pain are related to pain and dysfunction. It is thought that FRR may also be altered in acute low back pain conditions. Zedka et al. (1999) examined the effect of hypertonic saline injection in the low back to induce acute LBP and measure subsequent EMG activity alterations. These authors found that there was indeed an absence of relaxation of the lumbar paraspinals during full flexion, but the generalizability of these findings are limited due to the small sample size (*n* = 5) and method of low back pain induction [6]. The need for further study of the FRR response in those with acute low back pain is indicated [5].

Consequently, the objective of this current study was to determine whether differences exist in the FRR between individuals experiencing significant acute muscular low back pain and those with minimal low back pain from a delayed onset muscle soreness (DOMS) protocol. Subjective current pain intensity ratings at 24 and 48 hours after DOMS were used to classify participants into pain groups. The primary hypothesis was that a differential response in FRR, a representation of muscular performance, would be found between participants who reported pain after the DOMS protocol compared to participants reporting minimal LBP. 

## 2. Materials and Methods

### 2.1. Participants

51 volunteers (31 women, 20 men; mean 22.1 years, SD 3.5; mean BMI 23.9, SD 4.5) read and signed an informed consent form approved by the University Institutional Review Board. Participants were excluded if they met any of the following criteria: previous participation in a conditioning program specific to trunk extensors, any current low back pain, any chronic medical conditions that may affect pain perception (e.g., diabetes, high blood pressure, fibromyalgia, headaches), kidney dysfunction, muscle damage, major psychiatric disorder, history of previous injury including surgery to the lumbar spine, renal malfunction, cardiac condition, high blood pressure, osteoporosis, liver dysfunction, or intervention for symptoms induced by exercise before the termination of their participation in the protocol. Additionally, participants were dropped from statistical analyses if they had missing data or reported pain at baseline unrelated to the low back.

### 2.2. Measures

All measures were taken at baseline (preexercise protocol) 24 and 48 hours after DOMS protocol.

#### 2.2.1. Pain Intensity in the Low Back

The pain visual analog scale (VAS) is a 100 mm line anchored at one end with “none” and at the other with “worst pain imaginable.” Participants rated their current pain intensity by placing a mark along the line. A previous study has indicated that the VAS is a valid ratio measure for pain intensity [7]. Two groups were formed by categorizing participants' reported current pain ratings. The group who reported having a current pain intensity rating of ≥15 mm/100 mm on the VAS at either 24 or 48 hours was categorized in the “pain” group. The group reporting <15/100 mm on the VAS for current pain from baseline to 24 or 48 hours was categorized in the “minimal pain” group. These groups were selected based on literature review which stated a change of 15 mm/100 mm provided minimal important change in pain in persons with heterogeneous causes and duration of low back pain, including those with acute LBP [8]. Evidence suggests that the higher the baseline severity of pain and disability, the larger the improvement (or change) has to be for the patient to perceive as a “clinically important” change [9]. Therefore in this sample, a threshold of 15 mm/100 mm was appropriate since all patients reported being pain-free at baseline.

#### 2.2.2. Range of Motion

Trunk flexion range of motion (ROM) in the sagittal plane and straight leg raise (SLR) ROM were measured as part of the Physical Impairment Index [10] using a standard inclinometer. The use of the inclinometer is a reliable and valid measurement tool for measurement of trunk ROM [11]. The average of three repetitions was recorded following the standardized measurement protocol.

#### 2.2.3. Flexion Relation Ratio

The flexion relaxation ratio (FRR) is a quantifiable measure of muscle performance. In a study comparing patients with LBP to healthy controls, the discriminate validity for the FRR was 93% sensitivity and 75% specificity, clearly discriminating patients with LBP from controls [12]. In this study FRR was collected using surface electromyography (sEMG).

#### 2.2.4. Surface Electromyography

**Figure 1:**
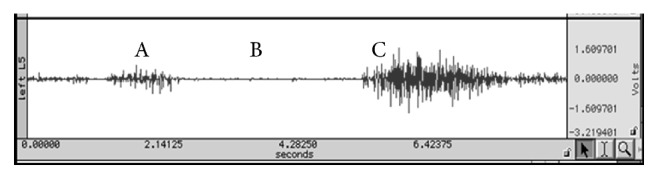
Sample of raw sEMG from the erector spinae on the left of the L5 lumbar level. A is active trunk flexion. B represents the point in the trunk range of motion where the lumbar paraspinals become myoelectrically silent and the passive structures such as the posterior spinal ligaments, discs, and fascia provide terminal flexion. Lastly, C represents the return from full trunk flexion.

Surface EMG data were collected from paraspinal muscles bilaterally. Electrodes were placed 2.5 cm lateral to the spinous processes of T12 and L5 [12]. Each recording electrode consisted of two silver-silver chloride 1 cm diameter electrodes embedded in an epoxy-mounted preamplifier system (×35) whose centers were spaced 2 cm apart. A reference electrode was attached to the medial aspect of the tibial shaft. Signals were band-pass filtered (20 Hz to 4 KHz) (Therapeutics Unlimited, Iowa City, Iowa, USA), and low-pass filtered (350 Hz) online. Processed sEMG was sampled online at a rate of 1,000 Hz (BIOPAC Systems, Goleta, CA, USA). Data were further processed by calculating the root mean square (RMS) over a 50 ms window of time during myoelectrical silence in full flexion and during trunk extension. A sample of the raw sEMG collected from the erector spinae is shown in Figure 1.

**Figure 2:**
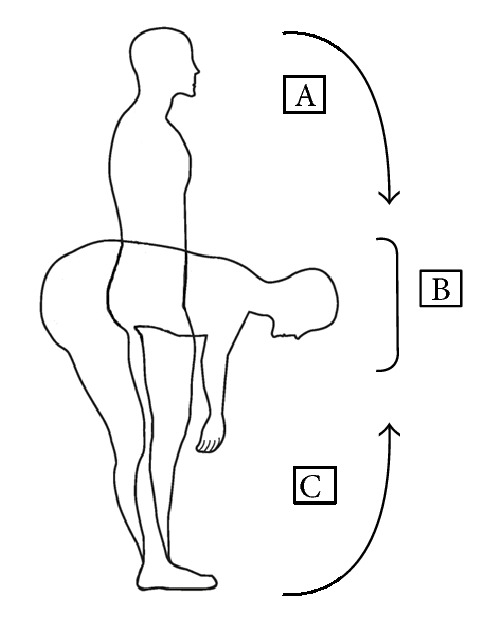
This figure represents the trunk motions involved in the flexion relaxation ratio. A is active trunk flexion. B represents the point in the trunk range of motion where the lumbar paraspinals are thought to become myoelectrically silent and the passive structures such as the posterior spinal ligaments, discs, and fascia provide terminal flexion. Lastly, C represents the return from full trunk flexion where lumbar paraspinals are myoelectrically active.

After electrodes were placed on the lumbar paraspinals, participants were asked to flex their trunk forward as far as they were able without bending their knees for a count of six, hold the final position for one second, and return to upright stance for a count of six [12]. One second of the RMS sEMG during the ascent phase was divided by the RMS sEMG during the one second pause between ascent and descent (myoelectrical silence), creating the flexion relaxation ratio. See Figure 2 for illustration.

### 2.3. Delayed Onset Muscle Soreness Protocol (DOMS)

All participants completed a warm-up session consisting of riding the stationary bicycle and passive stretching of the lower extremities. Each participant performed a baseline isometric test of total torque using a MedX lumbar extension exercise machine. Isometric testing was performed from the participants' maximal seated trunk flexion and every 12° till maximal seated trunk extension. The repeatability of isometric torque production is well established in participants without pain [13] and groups of patients with LBP [14]. 

To perform the dynamic fatiguing exercise bout, the participants were seated and restrained in a MedX lumbar extension exercise machine to insure proper use of lumbar extensors. Participants performed repetitions until self-reported limit using a weight load equal to approximately 80% of the peak torque measured during the isometric test. Each repetition was performed through the full available range of motion (ROM). Repetitions of both exercises continued until the patient reported being unable to move through a full ROM achieving volitional fatigue. At the end of the exercise set, the isometric torque test was performed again. Participants repeated the sequence of dynamic exercise and static testing until total measured torque decreased to 50% of the baseline torque measurement from the first isometric test. Participants were instructed not to initiate any pain relieving medication or apply any palliative intervention to the lumbar spine after exercise protocol to insure proper measurement of muscular pain. This method of muscle fatigue was found to sufficiently cause muscular low back pain. Details regarding pain and disability outcomes using this model are presented elsewhere [15].

### 2.4. Analysis

Pain groups were compared for differences at baseline using *t*-tests for continuous variables and Chi-squared for categorical variables. Associations among change in FRR at 48 hrs and loss of range of motion at 48 hrs were assessed by testing the zero-order correlations. 

To test the hypothesis that acute onset muscular LBP would influence FRR, separate mixed repeated measures analyses of covariance (ANCOVA) models were built (between—pain group, within—time (3 levels); covariates—age, gender, baseline trunk ROM) for the average of the spinal levels at which sEMG was collected. Specifically we tested interaction effects for time and pain groups; we expected the FRR would be reduced indicating increased muscular activity in relaxation period for the pain group but not the minimal pain group at 48 hrs after DOMS protocol. Separate ANCOVA models were also built to assess changes in trunk ROM and SLR ROM between pain groups (between—pain group, within—time (3 levels); covariates—age, gender). Type 1 error was maintained at 5% and all analyses were performed using IBM SPSS 20.

## 3. Results

The reported current pain intensity at 24 and 48 hours for the 51 participants ranged from 0 to 68 mm on the VAS. The subjects that reported pain greater than 0/100 mm on the VAS at baseline were dropped from the analysis, leaving 42 participants to be analyzed. Twenty-one participants reported a current pain intensity of (≥15/100 mm) at 24 or 48 hours and 21 participants reported a pain intensity of <15/100 mm. Groups did not differ at baseline in age, gender, SLR, or FRR but differed in trunk ROM (*t* = 2.24, *P* = 0.031), where the pain group had slightly decreased trunk ROM compared to the minimal pain group (pain group = 104.29 ± 12.80, minimal pain group = 112.83 ± 13.76). See Table 1 for descriptive statistics.

 Correlations revealed a negative correlation between age and sex (*r* = −0.32, *P* = 0.04), a negative correlation between change in pain and change in trunk flexion ROM (*r* = −0.375, *P* = 0.014), and a positive correlation between change in SLR and FRR at 48 hours (*r* = 0.494, *P* = 0.003). See Table 2 for correlations.

### 3.1. Flexion Relaxation Ratio

The mean FRR was decreased at 24 hours in both groups and decreased in the pain group only at 48 hours; however none of these time effects were found significant (*F*_2,29_ = 0.43, *P* = 0.958). There was no effect of pain group (*F*_1,29_ = 0.054, *P* = 0.818) or baseline trunk flexion ROM (*F*_1,29_ = 0.951, *P* = 0.338) on changes in FRR nor were there any significant two-way interactions. 

### 3.2. SLR ROM

SLR ROM was decreased at 24 and 48 hours after DOMS in both groups. Analysis of changes in average SLR ROM showed no main effect of pain group (*F*_1,38_ = 3.51, *P* = 0.057) nor any significant two-way interactions. 

### 3.3. Trunk ROM

Trunk ROM was decreased at 24 and 48 hours after DOMS in both groups. There was a main effect of pain group on changes in trunk ROM over time (*F*_1,38_ = 7.21, *P* = 0.011), where the pain group had greater losses in trunk flexion ROM than the minimal pain group, but no two-way interactions were found.

## 4. Discussion

 Contrary to our main hypothesis that FRR would be affected by acute onset muscle pain from a DOMS protocol, the presence of pain (current pain report of ≥15/100 mm on VAS) did not affect muscular activation during active trunk flexion and relaxation as hypothesized. These findings suggest that changes in FRR may not be significantly affected by acute muscular low back pain from a DOMS protocol.

Past research has supported that changes in FRR are responsive in the presence of chronic LBP, whereas our study did not find that FRR was responsive in the presence of acute muscular LBP. The current study used a DOMS protocol to induce acute LBP; past studies reporting the responsiveness of FRR to pain had notable methodological differences from our study which may explain the difference in reported results. Other published studies have examined the changes in FRR over a timeframe other than 48 hours which is when peak pain from DOMS is thought to occur. Additionally, other published studies use noxious stimulus to induce LBP rather than a muscular pain inducing protocol. The DOMS protocol induces pain of a muscular origin, which is a common cause of acute LBP. Therefore the findings of study support that changes in pain from a muscular origin may not influence FRR as significantly as pain generation by other methods or in a different time frame.

Furthermore, several models have been proposed to explain aberrant muscle activity in subjects experiencing LBP. One theory proposes that LBP may be the result of muscle asymmetries. Literature suggests that the paraspinal muscles of patients with LBP act submaximally [16, 17] and there is reduced activity during trunk movements [18, 19]. Also, Hides et al. (1994) suggest that arthrogenic muscle inhibition is likely in the paraspinal muscles in the presence of LBP. Such changes can potentially affect the sEMG measured in these subjects and patients. In this study, our findings did not support changes in muscle activation in the presence of acute muscular low back pain. 

Correlations revealed a positive relationship between changes in SLR ROM and changes in FRR at 48 hours, meaning the greater the loss of SLR ROM, the lower the FRR ratio (less lumbar muscle relaxation). Additionally, there was not a main effect found for pain group on changes in SLR ROM. Therefore this finding suggests that SLR ROM, and potentially hamstring flexibility, adversely affects achieving muscle relaxation. Moreover, the presence of acute low back pain does not seem to affect SLR ROM, but this may be affected in persons with chronic low back pain. This finding is consistent with the understanding that hamstring flexibility can be restricted in those experiencing nonspecific low back pain and may affect muscle activation patterns [20]. 

Additionally, literature suggests flexion relaxation and therefore FRR is likely a function of trunk ROM. Neblett et al. (2003) suggest failure to reach adequate lumbar flexion ROM may result in failure to cause flexion relaxation. In this study, no associations were found between change in trunk flexion ROM and FRR at 48 hours nor did baseline trunk ROM have an effect on changes in FRR. But there was a main effect of pain group on changes in trunk flexion ROM. Our findings suggest that decreased low back extensor flexibility may make an individual more susceptible to developing DOMS from low back exercises and potentially have greater changes in trunk flexion ROM in the presence of pain. The presence of acute low back pain may impact functional mobility, such as bending over (trunk flexion) in persons with acute low back pain in the absence of producing muscle corresponding activation changes (FRR) seen in persons with chronic LBP.

Limitations in the current study include the relatively young sample and specificity of findings to those with acute low back pain from a muscular origin. Past studies have supported using the FRR as an indicator of clinical status in those with chronic low back pain, but have not been validated in those with acute low back of a muscular origin. 

## 5. Conclusions

Our results suggest that the FRR is not responsive to short duration acute low back pain of a muscular origin from a DOMS protocol. These findings suggest the utility of the FRR may be limited in patients with acute LBP and may be more useful in patients with longer standing low back pain. Consequently, based on the results of this study, FRR as a diagnostic tool may be most useful in persons with chronic low back pain and is limited in those with acute low back pain of muscular origin. 

## Figures and Tables

**Table 1:** Clinical Measures by pain group (mean (SD)).

Pain group	Time	Pain score (100 mm VAS)	Trunk flexion (degrees)∗	FRR (ratio)	Straight leg raise (degrees)
	Baseline	0	104.52 (12.93)	22.75 (30.84)	97.69 (11.68)
Pain (*n* = 21)	24 hours	23.19 (14.06)	101.67 (11.45)	15.75 (21.42)	86.14 (13.83)
	48 hours	27.0 (17.10)	95.38 (22.81)	8.44 (6.96)	76.54 (28.4)

	Baseline	0	113.76 (13.77)	13.00 (10.77)	103.73 (14.74)
Minimal pain (*n* = 21)	24 hours	5.48 (5.43)	109.76 (13.19)	12.90 (15.93)	88.95 (13.10)
	48 hours	5.14 (5.60)	110.14 (14.18)	14.74 (21.79)	89.69 (12.32)

^*^Mean difference between pain groups is significant at the 0.05 level.

**Table 2:** Correlation of variables at baseline.

	Age	Sex	Change in FRR at 48 hours	Change in straight leg raise	Change in trunk flexion	Change in pain report
Age	—	—	—	—	—	—
Sex	−0.32∗	—	—	—	—	—
Change in FRR at 48 hours	−0.05	−0.05	—	—	—	—
Change in straight leg raise	0.09	−0.03	0.49∗∗	—	—	—
Change in trunk flexion	−0.06	−0.05	0.09	0.18	—	—
Change in current pain report	−0.19	0.02	−0.67	−0.20	−0.37∗	—

^*^Correlation is significant at the 0.05 level.

^**^Correlation is significant at the 0.01 level.
